# Effectiveness of the German disease management programs: quasi-experimental analyses assessing the population-level health impact

**DOI:** 10.1186/s12889-021-12050-7

**Published:** 2021-11-15

**Authors:** Jacob Burns, Christoph Kurz, Michael Laxy

**Affiliations:** 1 Institute for Medical Information Processing, Biometry and Epidemiology, TU München, Marchioninistrasse 17, 80336 Munich, Germany; 2 Institute for Medical Information Processing, Biometry and Epidemiology, LMU Munich Marchioninistrasse 17, 80336 Munich, Germany; 3 Munich School of Management and Munich Center of Health Sciences, LMU Munich, Munich, Germany; 4 Pettenkofer School of Public Health, Munich, Germany; 5 Institute of Health Economics and Health Care Management, Helmholtz Zentrum München, German Research Center for Environmental Health (GmbH), Ingolstädter Landstraße 1, 85764 Neuherberg, Germany

**Keywords:** Disease management, Program evaluation, Diabetes mellitus, type 2, Coronary disease, Mortality

## Abstract

**Background:**

In 2002–2003 disease management programs (DMPs) for type 2 diabetes and coronary heart disease were introduced in Germany to improve the management of these conditions. Today around 6 million Germans aged 56 and older are enrolled in one of the DMPs; however, their effect on health remains unclear.

**Methods:**

We estimated the impact of German DMPs on circulatory and all-cause mortality using a synthetic control study. Specifically, using routinely available data, we compared pre and post-intervention trends in mortality of individuals aged 56 and older for 1998–2014 in Germany to trends in other European countries.

**Results:**

Average circulatory and all-cause mortality in Germany and the synthetic control was 1.63 and 3.24 deaths per 100 persons. Independent of model choice, circulatory and all-cause mortality decreased non-significantly less in Germany than in the synthetic control; for the model with a 3 year time lag, for example, by 0.12 (95%-CI: − 0.20; 0.44) and 0.22 (95%-CI: − 0.40; 0.66) deaths per 100 persons, respectively. Further main analyses, as well as sensitivity and subgroup analyses supported these results.

**Conclusions:**

We observed no effect on circulatory or all-cause mortality at the population-level. However, confidence intervals were wide, meaning we could not reject the possibility of a positive effect. Given the substantial costs for administration and operation of the programs, further comparative effectiveness research is needed to clarify the value of German DMPs for type 2 diabetes and CHD.

**Supplementary Information:**

The online version contains supplementary material available at 10.1186/s12889-021-12050-7.

## Background

Diabetes and cardiovascular disease are two highly prevalent diseases that pose a large health and cost burden on patients and society. In Germany around 40% of deaths and around 18% of annual health care expenditures are attributable to these two conditions, with coronary heart disease (CHD) being the major contributor to the burden of cardiovascular disease [1, 2]. The adequate management of diabetes and CHD is highly complex and challenging for health care providers, patients and the health care system as a whole. It requires regular check-ups, close monitoring of blood pressure, cholesterol and blood glucose, active patient involvement in managing the disease between check-ups and good coordination of care between general practitioners, nurses, diabetes educators, specialists, and hospitals [3, 4]. Various disease management programs (DMPs) have been developed to address these aspects, and many of them have been shown to be effective in improving intermediate outcomes, such as control of blood glucose, hemoglobin and lipid levels [5, 6].

In 2002/2003 DMPs for type 2 diabetes and CHD were rolled out nationwide in Germany in the statutory health insurance (SHI) system, with the aim of reinforcing guideline care and improving quality of care for the chronically ill [7]. Since their implementation the proportion of patients enrolled in the German DMPs for type 2 diabetes and CHD rose to more than 50% of patients with each respective indication, and today around 6 million people in total are enrolled in the two DMPs [8]. However, despite its high enrollment and estimated annual program costs of around 870€ million (assuming 6 million enrollees and annual DMP administration costs of 145€), the effectiveness of the German DMPs for diabetes and CHD has remained uncertain. A systematic review of the effectiveness of the German DMP for type 2 diabetes, by Fuchs et al., found a positive effect across most studies on process parameters, such as the proportion of patients receiving annual check-ups and indicated medication. Furthermore, the few studies that analyzed mortality and survival also found large positive effects for the type 2 diabetes DMP [9–12]. Authors further discussed that, due to spillover-effects that might occur when physicians also treat non-enrollees according to DMP standards, the net effect of the intervention might even be underestimated in studies that compare DMP enrollees and controls [13]. However, all of these studies are based on non-randomized comparisons using data from insurance claims, cohort studies or registries and use regression, matching or propensity score methods to control for selection bias. As those approaches do not balance unobservable factors it is possible that the reported effects on health outcomes and mortality might be explained by differences in unobserved characteristics between DMP enrollees and non-enrollees [14, 15]. Those methodological limitations in evaluations of the DMPs have been discussed previously by other authors [16, 17].

In this study, we use a different approach, adopting a population-level health perspective, in evaluating the effectiveness of German DMPs for type 2 Diabetes and CHD in reducing mortality. We apply a quasi-experimental study design, specifically a synthetic control (SC) study, in assessing whether the 2002/2003 introduction of the German type 2 diabetes and CHD DMPs led to reduced population-level mortality relative to other European countries. This approach allowed us to estimate the net effect of the German DMPs on mortality, while accounting for potential spill-over effects and avoiding selection bias.

## Methods

### Intervention and context

The DMPs for type 2 diabetes and CHD were rolled out through the SHI system in 2002 and 2003, respectively [7]. The SHI is a system of non-profit health insurance companies insuring around 88% of the German population. In contrast to DMPs in the United States and other countries, which often focus on high risk patients, German DMPs target all patients with the disease and are characterized by a high degree of homogeneity. The key contents of the German DMPs for diabetes and CHD are enforcement of medication therapy, enhanced patient activation and self-management education, continuity of care according to current guidelines and the use of information technology systems for routine documentation/benchmarking [18]. Whereas the enrollment of patients in those programs was initially incentivized by a large risk surcharge, since 2009 physicians and the health insurance receive a flat rate premium of 125€ and 20€ per year per DMP enrollee from the central ‘German Health Fond’ [7, 19, 20]. Since their implementation, the proportion of patients enrolled in the German DMPs for type 2 diabetes and CHD rose to more than 50% of individuals with those respective indications, to around 6 million people (Fig. S1 in Additional file 1). Currently, 87 and 94% of the enrollees in the type 2 diabetes and CHD DMPs, respectively, are 56 years and older, meaning that around 5 million of the 26 million Germans (around 20% of the population) aged 56 years and older are enrolled in one of these two DMPs [8, 21].

### Study design

In order to assess the effectiveness of the German DMPs we conducted a SC study [21], where we compared circulatory system-related and all-cause mortality rates before and after DMP implementation in the German elderly population with those of other European countries. We concentrated on circulatory and all-cause mortality, because, in the long-term, it can be expected that better care processes lead to better intermediate clinical outcomes and translate to longer survival. Furthermore, as most of the targeted care in the German DMPs targets cardio-metabolic care processes (blood pressure, lipids, HbA1c), we assumed that the effect of the DMPs would be strongest for circulatory mortality.

The SC study is a quasi-experimental study similar to a difference-in-differences study, but it allows for the construction of a control group in instances where there are several sites to choose from, but no clear rationale for choosing the most appropriate control site. Specifically, a SC study compares changes in areas receiving the intervention with changes in a weighted average of control areas that provides the most similar comparison, with respect to the pre-intervention outcome trend and a pre-defined set of covariates [22]. Through the estimation of this counterfactual, i.e. what would have happened had the intervention not been implemented, one can account for existing secular trends, as well as for potential changes in the outcome not associated with the intervention occurring on a larger geographical scale [18].

### Data

**Outcomes:** We obtained data on mortality and population for the years 1998–2014 from the World Health Organization’s (WHO) Cause of Death Query (CoDQL) online platform for several European countries: Austria, Belgium, Bulgaria, Croatia, Cyprus, the Czech Republic, Denmark, Estonia, Finland, France, Germany, Ireland, Italy, Latvia, Lithuania, Luxembourg, Malta, the Netherlands, Poland, Portugal, Romania, Slovakia, Slovenia, Spain, Sweden, Switzerland and the United Kingdom. We excluded Greece and Hungary because valid outcome data were not available. For included countries, we extracted the overall and sex/age stratified annual numbers of deaths due to diseases of the circulatory system (ICD-10: I00-I99) and all-cause deaths. By combining this mortality data with overall and sex/age stratified annual numbers of population sizes, we calculated overall and age-stratified mortality rates.

**Covariates:** We further identified demographic (age, sex), economic (per capita gross domestic product (GDP), unemployment rate, health care expenditure in % of GDP), clinical (prevalence of diabetes, hypertension and obesity) and behavioral (smoking and alcohol consumption) factors that are potentially associated with circulatory and all-cause mortality and for which data was available for the respective years and countries. Data on these factors were extracted from the OECD, the World Bank, the NCD Risk Factor Collaboration and the WHO (Table S1 in Additional file 1). As data on smoking and alcohol consumption were unavailable for Croatia, Estonia, Malta and Romania for the entire study period, we excluded these countries from the analysis. Where intermittent data were missing for a covariate, we imputed these values using linear interpolation using the country’s previous and next annual value.

### Statistical analyses

#### Main analyses

In conducting a SC study one must define an intervention site, which is exposed to the intervention after its implementation, and a synthetic control donor pool, which should not be exposed to the intervention. In our study, Germany served as the intervention site and several European countries as the synthetic control donor pool. To ensure that results were unbiased by similar interventions, we conducted a series of non-systematic searches to identify interventions implemented in European countries that may have influenced the population-level diabetes and/or CHD rates from 2003 to 2009. We first checked the Cochrane Database of Systematic Reviews and Google Scholar for systematic reviews of such interventions. Additionally, for studies evaluating DMPs cited above, we used Google Scholar to identify studies subsequently citing these studies. Studies evaluating potentially relevant interventions were identified in some countries, however, in almost all cases these evaluated small-scale interventions rather than population-level interventions. According to these literature searches, the only country that introduced a nationwide program to improve the quality of chronic care was England, in which a pay for performance scheme was introduced in 2004. As an evaluation indicated that this program improved diabetes care [23], we excluded the UK from our analyses.

The SC group was created from the donor pool based on the pre-intervention outcome trend, as well as a set of pre-defined potentially important covariates, i.e. those described above. These aspects were weighted within the donor pool to best match the pre-intervention outcome trend in Germany, and to create the post-intervention counterfactual, i.e. how the outcome trend would have continued in Germany had the DMPs not been implemented. We calculated the treatment effect of interest, the average treatment effect on the treated (ATT), by estimating counterfactuals for Germany at each time point using control group information based on a linear interactive fixed effects model that incorporates unit-specific intercepts interacted with time-varying coefficients [22]. This treatment effect is the difference between the observed series, i.e. the post-intervention outcome trend observed in Germany, and the synthetic control time series, i.e. the post-intervention counterfactual series. Given the progressive enrollment of the German DMPs discussed above, and the resulting uncertainty regarding at which point they may have affected the mortality at the population level, we modeled the intervention at three different time points: immediately after implementation in 2003, after four years of enrollment in 2006, and after seven years of enrollment in 2009. As almost 90% of DMP participants are 55 years or older we restricted the main analysis to people in the following age groups: 55–64, 65–74 and 75 and older.

#### Subgroup and sensitivity analyses

To explore whether any differential effects were masked by the use of an aggregated age group of 55 years and older, we conducted the SC analysis for both outcomes, circulatory and all-cause mortality, in age subgroups, including ages 55–64, 65–74 and 75 and older. Additionally, to assess whether changes in cardiovascular and all-cause mortality across all ages, not just in the elderly, were influencing the study results, we repeated the main analyses for a younger age group, 20–54 years, because no effect, or at most a minimal effect, due to the intervention would be expected in this age group. This approach is similar to a difference-in-difference-in-differences estimate in which, given a real effect, we would expect differences in mortality trends in exposed populations (people aged 56 and older) but no differences in mortality trends in unexposed populations (people aged 55 and younger).

To ensure that no single control country was driving the results related to the DMP effects (for example if an unknown and effective population-level diabetes program had been implemented in a specific country in 2003), we conducted a series of leave-one-out analyses. Specifically, we conducted the main analyses repeatedly, each time removing a single country from the control donor pool.

All data processing and analyses were conducted using R version 3.3.2. The synthetic control analyses were conducted using the Generalized Synthetic Control Method (gsynth) package [24].

## Results

### Construction of synthetic control

As described above, in creating the counterfactual, pre-intervention outcome trends in the donor pool, as well as similarities with respect to other key covariates were considered. Fig. S2 in Additional file 1 illustrates the trends in circulatory mortality (top panel) and all-cause mortality (bottom panel) in Germany as well as the rest of the European countries from 1998 to 2014; it is clear that both circulatory and all-cause mortality reduced substantially in Germany and across Europe during the study period.

Table 1 provides the weights calculated for each covariate and country, for each of the two outcomes and three intervention time points. Among the covariates, the prevalence of diabetes was assigned the heaviest weight across models, while age structure and the prevalence of obesity and hypertension also contributed to a lesser extent. The other covariates, including GDP and unemployment, were either not selected or were assigned very little weight across all models. Regarding the countries, each country was assigned a weight, which can be interpreted as follows. A country with a pre-intervention mortality rate similar to Germany, e.g. the Czech Republic was assigned a weight relatively close to 1. Those countries with a much higher pre-intervention mortality rate than Germany, e.g. Latvia, were assigned a positive weight close to 0, while those countries with a much lower pre-intervention mortality rate than Germany, e.g. the Netherlands, were assigned a negative weight, close to 0.

**Table 1:** Model coefficients for the covariates and synthetic control weights for countries

Mortality category	Circulatory			All-cause		
Intervention time	2003	2006	2009	2003	2006	2009
**Covariables**						
Age	−4.02 (1.94)	− 4.02 (1.94)	−4.00 (2.05)	−8.70 (2.79)	−8.68 (2.65)	− 8.68 (2.69)
Sex	–	–	–	–	–	–
GDP per capita	0.00 (0.00)	0.00 (0.00)	0.00 (0.00)	0.00 (0.00)	0.00 (0.00)	0.00 (0.00)
Unemployment	0.00 (0)	0.00 (0.00)	0.00 (0.00)	−0.01 (0.01)	− 0.01 (0.01)	0.01 (0.02)
Health care expenditure	–	–	–	–	–	–
Smoking Prevalance	0.01 (0.01)	0.01 (0.01)	0.01 (0.01)	0.01 (0.01)	0.01 (0.02)	0.01 (0.01)
Alcohol consumption	–	–	–	–	–	–
Obesity prevalance	−2.25 (17.34)	−2.25 (17.50)	− 2.29 (18.33)	−8.72 (22.67)	−8.75 (22.58)	−8.75 (22.66)
Diabetes prevalance	15.41 (19.00)	15.41 (18.74)	14.74 (18.70)	13.89 (20.63)	13.89 (20.71)	13.89 (20.99)
Hypertension prevalance	−3.64 (6.36)	−3.64 (6.32)	−3.56 (6.63)	−5.58 (7.42)	−5.57 (7.73)	−5.57 (7.65)
**Countries**						
Belgium	−0.40	−0.41	−0.44	0.19	−0.27	− 0.13
Czech Republic	0.88	0.89	1.21	−0.23	0.66	0.89
Denmark	0.65	0.66	0.53	−0.64	0.43	0.15
Finland	−0.21	−0.22	− 0.05	0.18	− 0.01	0.18
France	−0.04	−0.04	0.08	0.16	0.29	0.56
Latvia	0.10	0.10	−0.42	−0.27	− 0.13	−0.46
Lithuania	−0.83	−0.85	−1.15	− 0.21	−1.11	−1.74
Luxembourg	−0.04	− 0.04	−0.06	0.94	−0.11	− 0.17
Netherlands	−0.16	− 0.17	−0.27	− 0.20	−0.16	− 0.20
Poland	0.28	0.29	0.51	−0.12	0.28	0.51
Slovenia	−0.20	−0.21	− 0.11	0.31	0.26	0.59
Spain	−0.28	−0.29	− 0.23	0.31	− 0.12	0.08
Sweden	0.53	0.54	0.57	−0.54	0.19	−0.08
Switzerland	−0.28	−0.28	− 0.18	0.13	− 0.20	−0.18

Summary of the model coefficients for all covariates (with standard error) and the weights donor pool countries, as calculated using the synthetic control approach, stratified for the two assessed mortality outcomes and the three modelled intervention time points. The standard error for model coefficients for the covariates are provided in parentheses

### Effectiveness of the GDMPs

**Fig. 1:**
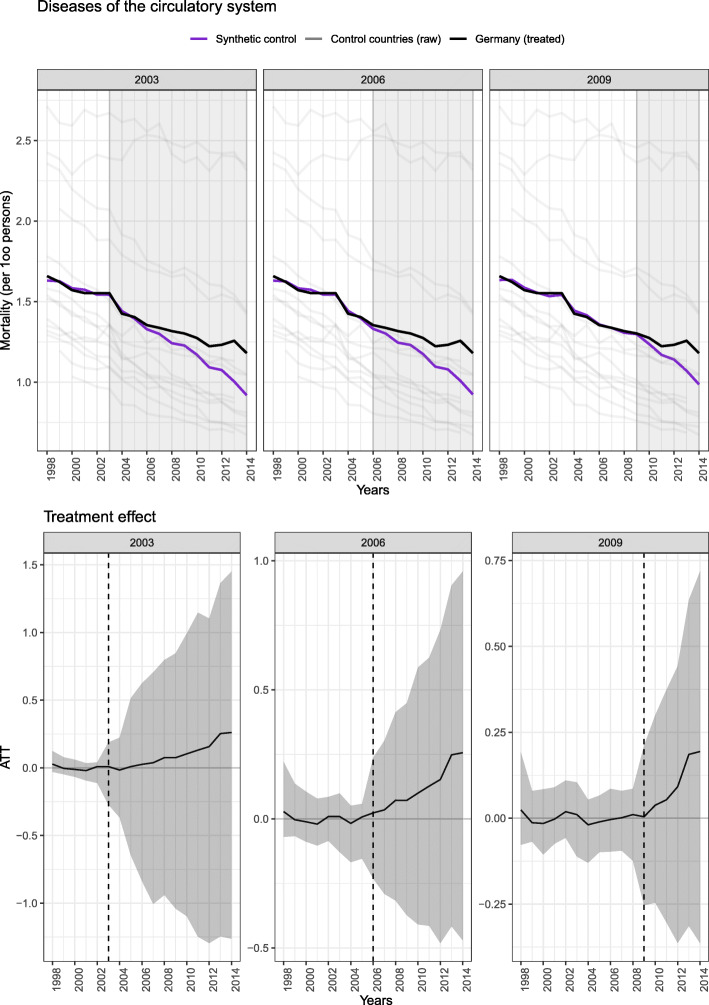
Effectiveness of the DMPs with respect to circulatory mortality in individuals aged 56 years and older in Germany compared to the synthetic control, expressed in mortality per 100 persons (top panel) and the ATT (bottom panel). Models considered the intervention time point as 2003 (left panels), 2006 (middle panels) and 2009 (right panels). Abbreviations: ATT: average treatment effect on the treated]

**Fig. 2:**
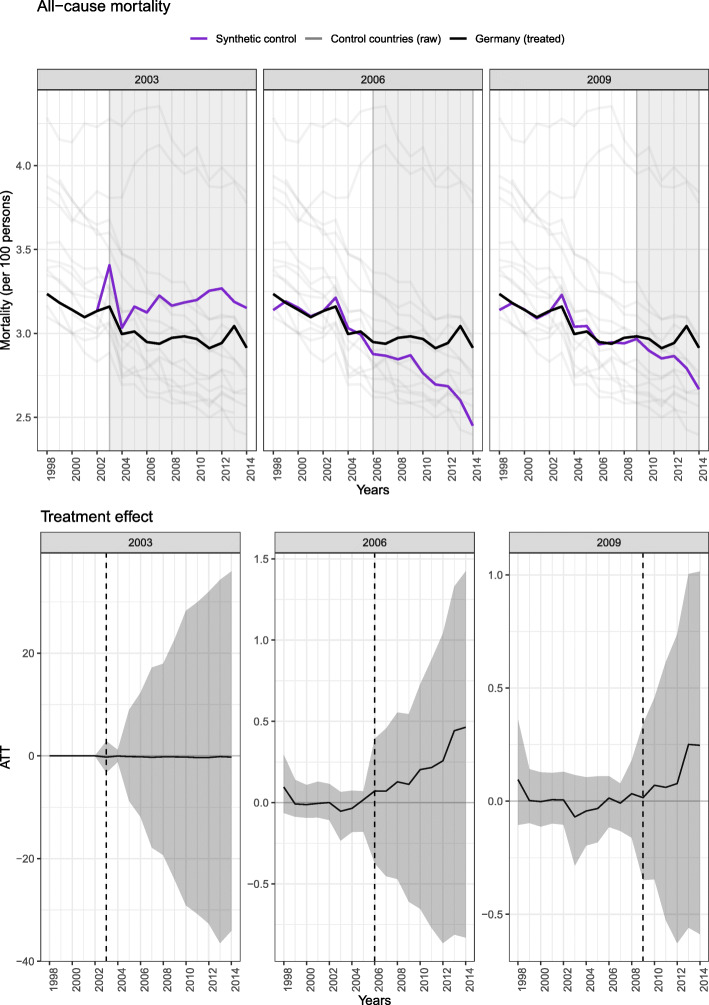
Effectiveness of the DMPs with respect to all-cause mortality in individuals aged 56 years and older in Germany compared to the synthetic control, expressed in mortality per 100 persons (top panel) and the ATT (bottom panel). Models considered the intervention time point as 2003 (left panels), 2006 (middle panels) and 2009 (right panels). Abbreviations: ATT: average treatment effect on the treated]

The effectiveness of the German DMPs in the population 55 years of age and above is summarized in Figs. 1 and 2. The top panels of both figures show the trends in mortality in Germany and the synthetic control, while the bottom panels show the ATT with 95% confidence interval (CI).

For circulatory mortality (Fig. 1), regardless of whether 2003 (left panel), 2006 (center panel) or 2009 (right panel) were considered the intervention point, the results are similar. For the pre-intervention period, the pre-intervention trend from the synthetic control matches that of Germany well. If the German DMPs were effective, we would expect the downward trend in Germany to become more pronounced relative to that of the synthetic control after the intervention point. As illustrated, however, this is not the case; the trend in the synthetic control relative to Germany became slightly more pronounced. As listed in Table 2, with 2003 as the intervention point, we observed an ATT of 0.09 (95% CI: − 0.63; 0.55), i.e. circulatory mortality decreased 0.09 deaths per 100 persons less in Germany than in the synthetic control over the study period. For the 2006 and 2009, we observed similar estimates: 0.12 (− 0.20; 0.44), 0.09 (− 0.19; 0.31), respectively. Based on the very small magnitude of each of these effects and the very wide confidence intervals, we did not observe any change in circulatory mortality associated with the German DMPs.

**Table 2:** Average treatment effect on the treated

Intervention time point	2003		2006		2009	
Mortality type	Est^a^	95-% CI	Est	95-% CI	Est	95-% CI
Circulatory	0.09	[−0.63; 0.55]	0.12	[−0.20; 0.44]	0.09	[−0.19; 0.31]
All-cause	−0.21	[−2.98; 2.80]	0.22	[−0.40; 0.56]	0.12	[−0.35; 0.53]

Summary of the ATT for both outcomes, circulatory and all-cause mortality, for the modelled intervention time points of 2003, 2006 and 2009

^a^Estimate (Est): annual death per 100 persons

For all-cause mortality (Fig. 2), we observed a very similar pattern when the years 2006 (center panel) and 2009 (right panel) were considered the intervention point. Here, after similar pre-intervention trends, the trend in the synthetic control became slightly more pronounced relative to Germany. The associated effects we observed were 0.22 (− 0.40; 0.56) and 0.12 (− 0.35; 0.53), respectively. When 2003 was considered the intervention point, however, the trend in Germany became slightly more pronounced relative to the synthetic control was seen, with an ATT of − 0.21 (− 2.98; 2.80). Once again, however, based the small magnitude of each of these effects and the broad confidence intervals, we did not observe any change in all-cause mortality associated with the German DMPs.

### Subgroup and sensitivity analyses

All subgroup analyses showed a similar pattern (Additional file 1, Table S2 and Figs. S3-S10. These included the individual subgroups that were aggregated in the main analysis, 55–64, 65–74 and over 75 years, as well as the younger subgroup not included in the main analysis, 20–54 years). Mortality developed in Germany and in the synthetic control group as would be expected, i.e. the lowest mortality in youngest subgroup and the highest mortality in the oldest subgroup. Regarding effectiveness, although trends generally became less stable and the synthetic control group matched Germany less well given the decrease in statistical power, the overall interpretation remained the same as for the main analyses: we did not observe any changes in either outcome associated with the German DMPs, regardless of intervention time point.

For the leave-one-out sensitivity analyses, we likewise observed results similar to the main analysis. For circulatory mortality (Additional file 1, Figs. S11), results were very similar with regard to both direction and magnitude. For all-cause mortality (Additional file 1, Figs. S12), results were similar overall, yet less stable; however, given the wide confidence intervals, none of the analyses would lead to a different interpretation of the effect of the DMPs.

## Discussion

German DMPs for type 2 diabetes and CHD belong to the biggest DMPs worldwide and administrative costs of those programs are high; however, their effectiveness remains unclear. In contrast to previous studies that analyzed the effect of DMPs using regression, matching or propensity score methods in German datasets, we applied a quasi-experimental study design to compare mortality trends in the German elderly population and mortality trends in other European countries before and after the introduction of the DMPs for DM2 and CHD. This allowed us to estimate not only the net effect of the German DMPs on mortality, but also effects from any potential spill-over of the interventions. Consistently across analyses and outcomes, we observed no clear effect associated with the DMPs in Germany, when compared to a synthetic control group comprising several European countries.

With the introduction of DMPs for type 2 diabetes and CHD in 2002 and 2003 every statutory insured patient with one of these conditions was eligible for participating in those programs. Given this ad-hoc introduction without a scientific testing phase, the short-term and long-term effectiveness and cost-effectiveness of the programs could not be tested in randomized study designs. Since then, several studies aimed to analyze the impact of DMPs on process and health outcomes using observational data. The majority of these focused on diabetes, and most are based on data from insurance claims, cohort studies or registries. Generally, the evidence from these studies suggests a positive impact of DMPs on process and health outcomes. A recent systematic review on the German DMPs for type 2 diabetes identified nine studies, which observed large improvements in mortality and morbidity associated with the DMPs. Stock et al. found a mortality reduction of 49%, Miksch et al. a reduction of 22% and Drabnik et al. a reduction of 49% [10–12]. However, each of the analyzed outcomes was only assessed by a single or a limited number of studies [9]. Since the publication of this review, further research based on observational data has pointed towards benefits associated with mortality and morbidity in the DMPs for COPD [19]. Most of the analyses in those studies, however, share methodological limitations which may have led to bias in the observed results. Each uses some method to account for selection bias, i.e. to account for differences in individuals enrolled in DMPs and individuals not enrolled in the DMPs. The methods applied to do so differ widely; some use propensity score matching based on several potentially relevant confounders [12, 25], while others more simple matching techniques [11, 26] or adjust for a small selection of variables, such as age and gender in regression analyses [27–29]. Across all of these studies, however, it is possible that further unobserved variables led to confounding in the observed effect estimates - a limitation also described by other authors commenting on methods for evaluating German DMPs [16, 17]. Furthermore, those studies based exclusively on German data within the SHI system were not able to assess the full effect of the DMP introduction, including potential spill-over effects due to general changes in the care patterns of general practitioners after the DMP implementation. Such effects may also have improved quality of care in DMP non-enrollees.

Our quasi-experimental approach allowed us to avoid the difficulties surrounding the observable and unobservable confounders at the individual level, and to estimate the full net effect of the DMP implementation at the population-level, including potential spill-over effects. These potential spill-over effects are important and should be included when evaluating a health policy from a comprehensive healthsystem perspective. We analyzed circulatory and all-cause mortality, two very distal endpoints in a potential logic model, but could not analyze process outcomes, intermediate clinical outcomes or patient relevant outcomes such as quality of life and patient satisfaction. However, long-term improvements in those process outcomes are expected to prolong life expectancy via improved control of blood sugar levels, hypertension and lipids.

Due to large confidence intervals in our effect estimates, our results cannot reject the possibility that DMPs have a positive effect, but make large positive effects on mortality as reported in the other studies very unlikely [10–12]. Given the results of this study on the effect of DMPs on circulatory and all-cause mortality, the investments for operating the two DMPs of approximately 870€ million may be questionable. Previous RCTs showed generally a positive effect of DMPs for diabetes on glycemic control, and process outcomes, but the success of these programs were often dependent on the design of the programs and the settings in which they were applied [5, 30]. Furthermore, the efficacy observed in these trials is likely to differ from the effectiveness observed in the real–world setting [31]. Many things that occur in the translation and implementation process of interventions into real world practice, including the rule of halves, could result into reduced or negligible effectiveness [1, 2]. Given all of this, future research that clarifies the impact of German DMPs on both short and long-term type 2 diabetes and CHD-related outcomes is highly warranted.

Our study has several strengths: We included best available data from several European countries in our analysis, which provided rich geographic variability in the construction of the synthetic control group. We considered multiple population-level aspects that may have differed between Germany and the control countries, including the prevalence of obesity, hypertension, smoking and alcohol consumption, the population age-structure, the gross domestic product (GDP) and the unemployment rate. This ensured that these potentially important confounders were accounted for in creating the synthetic control group. The use of time-series data from seventeen years, as opposed to a simple pre-, post-intervention analysis, also ensured that unobserved time-varying confounders were accounted for (Boutell 2018). Our results also show that the applied approach provided a well-matched synthetic control group with respect to the pre-intervention outcome trends. In addition, our various sensitivity analyses considering a later intervention start and comparing results in various exposed and non-exposed age groups show that our methods and results are robust towards changes in key assumptions.

Our approach was not without limitations, and it is important to consider these in interpreting the results. As described above, only 5–6 million out of the 25 million Germans over the age of 55 years are enrolled in the DMPs for type 2 diabetes or CHD, which is around 20% of this demographic strata. Our analysis, however, does not distinguish between enrollees and non-enrollees. This means that if, for example, the DMPs reduced mortality relatively by 20% without the existence of any spillover effects the maximal effect we could detect with our QE approach is a reduction of 4% in mortality. The large confidence intervals around our ATT estimate suggest that even mortality reductions of 20–50% as reported by previous studies could not be rejected with this approach [10, 11]. The relatively low power is related to the limited number of comparison countries and years of data. Furthermore, in our analysis where we took 2003 as intervention start, we included only four pre-intervention data points; conducting a synthetic control analysis based on this limited number of data points may lead a synthetic control group that is not well-comparable to the intervention group. However, our results suggest that the pre-intervention outcome trend in Germany was quite stable, and the synthetic control group matched this well. We have included a selection of covariates that are critical population-level determinants of circulatory and all-cause mortality; however, it is possible that this is not a comprehensive list, and that other variables should have been included in the analysis. Finally, mortality outcomes lie at the distal position of the causal pathway of the DMPs; shorter-term outcomes such has improved management, quality-of-life and patient satisfaction are also important for individuals and in aggregate from a public health perspective, yet are not captured in this study.

## Conclusion

In this quasi-experimental study applying a synthetic control design and analysis, we observed no effect of the German DMPs for type 2 diabetes and CHD on all-cause or circulatory mortality at the population-level. This pattern was consistent across all main, subgroup and sensitivity analyses. Confidence intervals were wide, and we could not reject the possibility of a positive effect. However, according to our analysis, the large mortality reductions as reported in previous studies are unlikely. Given the substantial costs for administration and operation of the programs, further comparative effectiveness research is needed to clarify the role of German DMPs for type 2 diabetes and CHD.

## Supplementary Information

**Additional file 1 Fig. S1.** Enrollment history for the German DMPs for type 2 diabetes and CHD. **Table S1.** Description of analyzed variables and the source of the data. **Fig. S2.** Trends in circulatory mortality (top panel) and all-cause mortality (bottom panel) in Germany as well as the rest of the European countries from 1998 to 2014. **Table S2.** The average treatment effects as well as the 95% confidence interval (CI), for each subgroup, each outcome and each intervention time point. **Fig. S3.** Subgroup analysis: disaggregated age (55–64; 65–74; 75+) for circulatory mortality, intervention point 2003. **Fig. S4.** Subgroup analysis: disaggregated age (55–64; 65–74; 75+) for circulatory mortality, intervention point 2006. **Fig. S5.** Subgroup analysis: disaggregated age (55–64; 65–74; 75+) for circulatory mortality, intervention point 2009. **Fig. S6.** Subgroup analysis: disaggregated age (55–64; 65–74; 75+) for all-cause mortality, intervention point 2003. **Fig. S7.** Subgroup analysis: disaggregated age (55–64; 65–74; 75+) for all-cause mortality, intervention point 2006. **Fig. S8.** Subgroup analysis: disaggregated age (55–64; 65–74; 75+) for all-cause mortality, intervention point 2009. **Fig. S9.** Sensitivity analysis: younger age group (20–54), where no or minimal effect is to be expected, for circulatory mortality. **Fig. S10.** Sensitivity analysis: younger age group (20–54), where no or minimal effect is to be expected, for all-cause mortality. **Fig. S11.** Sensitivity analysis: leave-one-country-out analyses for CHD mortality. **Fig. S12.** Sensitivity analysis: leave-one-country-out analyses for all-cause mortality.

## Abbreviations

ATT: average treatment effect on the treated
CHD: coronary heart disease
CI: confidence interval
CoDQL: Cause of Death Query
DMPs: Disease Management Programs
GDP: gross domestic product
ICD: International Classification of Diseases
OECD: Organisation for Economic Co-operation and Development
SC: synthetic control
SHI: statutory health insurance
WHO: World Health Organization
