# Correction to: Effectiveness of the German disease management programs: quasi-experimental analyses assessing the population-level health impact

**DOI:** 10.1186/s12889-021-12299-y

**Published:** 2021-12-07

**Authors:** Jacob Burns, Christoph Kurz, Michael Laxy

**Affiliations:** 1 Department of Sport and Health Sciences, Technical University of Munich, Georg-Brauchle-Ring 60/62, 80992 Munich, Germany; 2 Institute for Medical Information Processing, Biometry and Epidemiology, LMU Munich, Marchioninistrasse 17, 80336 Munich, Germany; 3 Pettenkofer School of Public Health Munich, Munich, Germany; 4 Munich School of Management and Munich Center of Health Sciences, LMU Munich, Munich, Germany; 5 Institute of Health Economics and Health Care Management, Helmholtz Zentrum München Research Center for Environmental Health (GmbH), Ingolstädter Landstraße 1, 85764 Neuherberg, Germany

**Correction to: BMC Public Health 21, 2092 (2021)**

**https://doi.org/10.1186/s12889-021-12050-7**

After publication of the original article [1] the authors noticed that several affiliations were incorrect.

The original affiliations were published as:

- 1 Institute for Medical Information Processing, Biometry and Epidemiology, TU München, Marchioninistrasse 17, 80336 Munich, Germany.
- 2 Institute for Medical Information Processing, Biometry and Epidemiology, LMU Munich Marchioninistrasse 17, 80336 Munich, Germany.
- 3 Munich School of Management and Munich Center of Health Sciences, LMU Munich, Munich, Germany.
- 4 Pettenkofer School of Public Health, Munich, Germany.
- 5 Institute of Health Economics and Health Care Management, Helmholtz Zentrum München, German Research Center for Environmental Health (GmbH), Ingolstädter Landstraße 1, 85764 Neuherberg, Germany.

The correction affiliations are:

- 1 Department of Sport and Health Sciences, Technical University of Munich Georg-Brauchle-Ring 60/62 80992 Munich, Germany
- 2 Institute for Medical Information Processing, Biometry and Epidemiology, LMU Munich Marchioninistrasse 17 80336 Munich, Germany
- 3 Pettenkofer School of Public Health Munich, Germany
- 4 Munich School of Management and Munich Center of Health Sciences, LMU Munich, Munich, Germany
- 5 Institute of Health Economics and Health Care Management, Helmholtz Zentrum München
- Research Center for Environmental Health (GmbH) Ingolstädter Landstraße 1 85764 Neuherberg, Germany

The original article has been updated.
