# Supplementary material for: Effectiveness of the German disease management programs: quasi-experimental analyses assessing the population-level health impact
Source: BMC Public Health. 2021 Nov 15;21:2092. doi: 10.1186/s12889-021-12050-7 (PMC8591814; doi:10.1186/s12889-021-12050-7)

Additional file 1

Figure S1

Enrollment history for the German DMPs for type 2 diabetes and CHD.

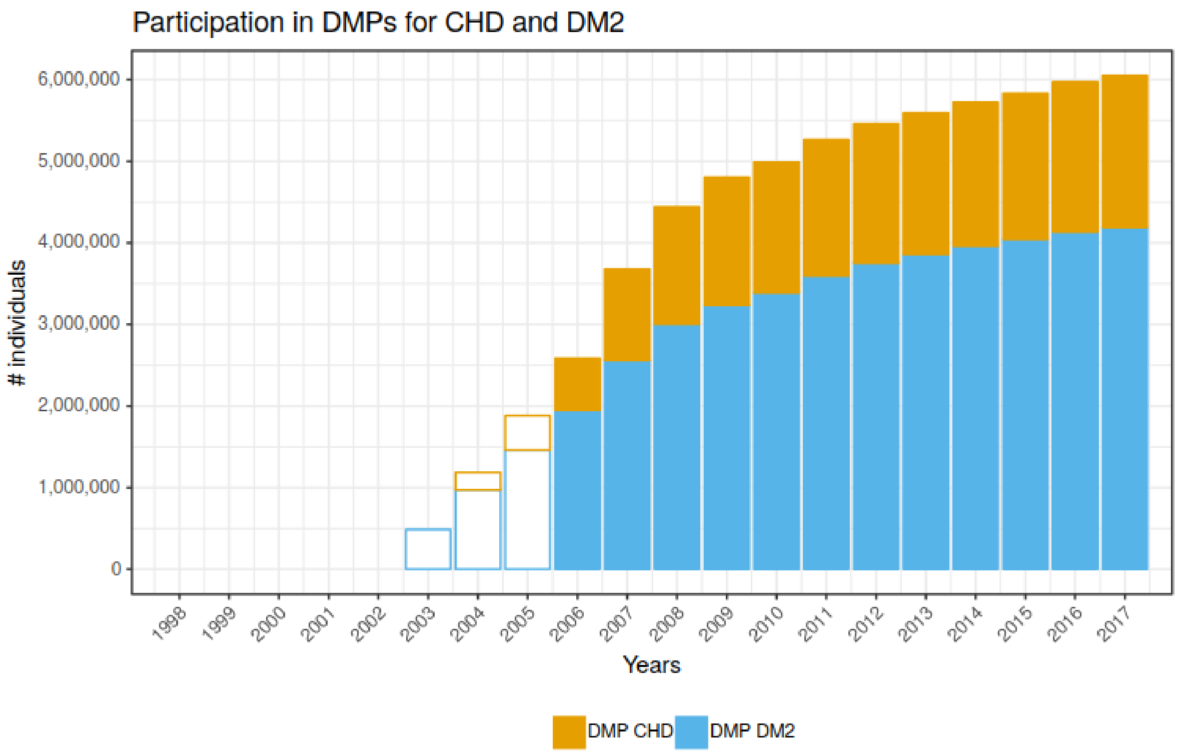

Table S1

Description of analyzed variables and the source of the data

Variables and data sources

Variable	Unit	Source	Source
Mortality	# of deaths	WHO	http://apps.who.int/healthinfo/statistics/mortality/causeofdeath_query/start.php
Population size	Total # alive	WHO	http://apps.who.int/healthinfo/statistics/mortality/causeofdeath_query/start.php
Age	20-54, 55-64, 65-74, 75+		http://apps.who.int/healthinfo/statistics/mortality/causeofdeath_query/start.php
Sex	men, women		http://apps.who.int/healthinfo/statistics/mortality/causeofdeath_query/start.php
GDP per capita	GDP/capita	World Bank	https://data.worldbank.org/
Unemployment	%	World Bank	https://data.worldbank.org/
Health care expenditure	% of GDP	World Bank	https://data.worldbank.org/
Smoking prevalance	% smokers	OECD	https://data.oecd.org/
Alcohol consumption	liter of alcohol/capita and year	OECD	https://data.oecd.org/
Obesity prevalance	% (age standardized)	NCD Risk Factor collaboration	http://ncdrisc.org/data-downloads.html
Diabetes prevalance	% (age standardized)	NCD Risk Factor collaboration	http://ncdrisc.org/data-downloads.html
Hypertension prevalance	% (age standardized)	NCD Risk Factor collaboration	http://ncdrisc.org/data-downloads.html

Figure S2

Trends in circulatory mortality (top panel) and all-cause mortality (bottom panel) in Germany as well as the rest of the European countries from 1998 – 2014

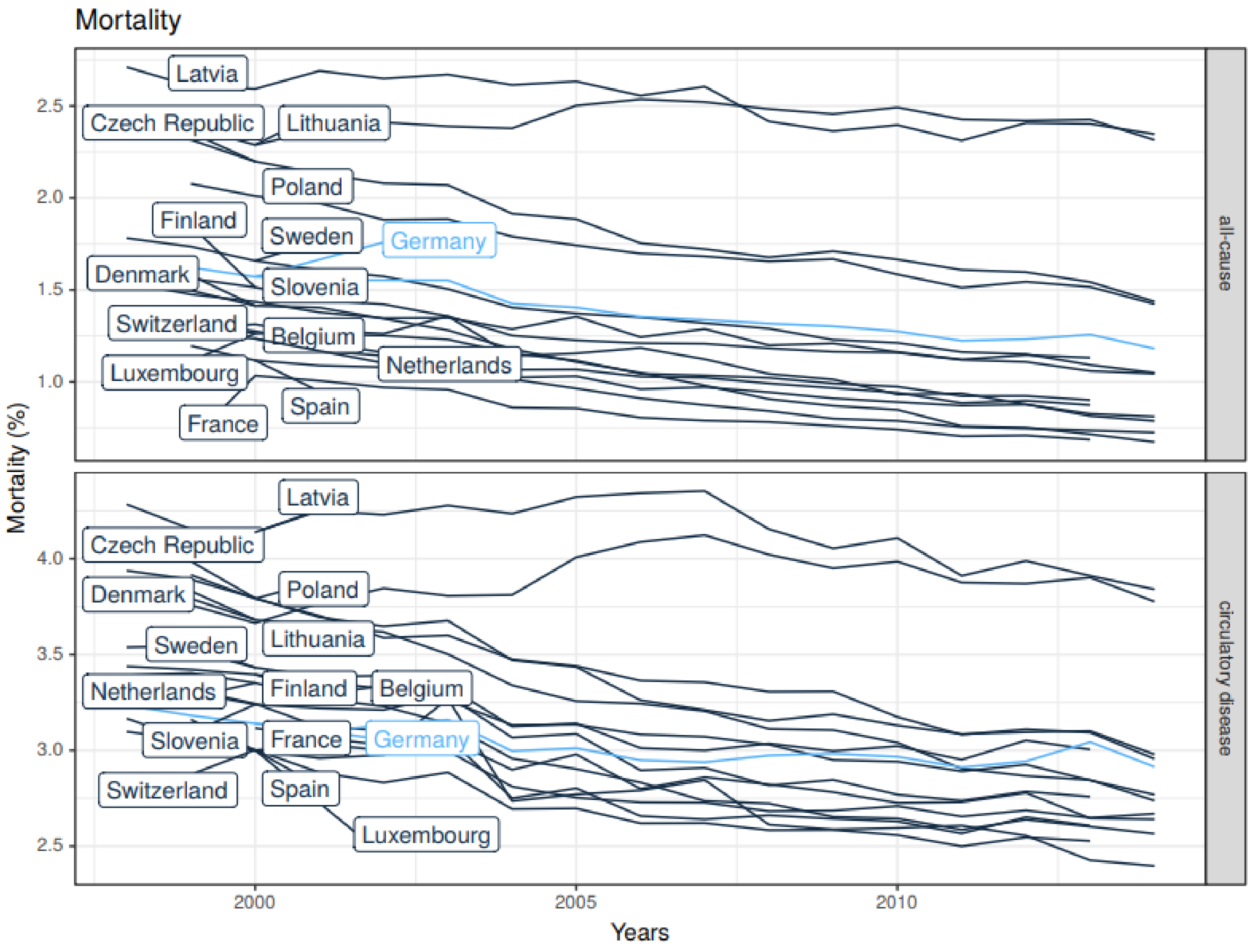

Table S2

The average treatment effects as well as the 95% confidence interval (CI), for each subgroup, each outcome and each intervention time point.

Intervention time		2003		2006		2009	
Age group	Mortality type	Est	95-% CI	Est	95-% CI	Est	95-% CI
55-64 years	Circulatory	-0.05	[-0.14; 0.04]	-0.03	[-0.11; 0.04]	-0.02	[-0.11; 0.04]
	All-cause	-0.03	[-0.41; 0.32]	-0.04	[-0.22; 0.09]	-0.04	[-0.24; 0.1]
65-74 years	Circulatory	-0.05	[-0.49; 0.59]	0.13	[-0.17; 0.35]	0.02	[-0.2; 0.18]
	All-cause	-0.27	[-1.01; 0.49]	0.24	[-0.42; 0.54]	0.18	[-0.22; 0.45]
75+ years	Circulatory	-0.3	[-1.09; 0.24]	0.04	[-0.63; 0.54]	-0.13	[-0.65; 0.34]
	All-cause	0	[-0.48; 0.48]	-0.04	[-0.64; 0.63]	-0.24	[-0.83; 0.53]
20-54 years	Circulatory	0	[-0.02; 0.01]	-0.01	[-0.03; 0.01]	0	[-0.02; 0.02]
	All-cause	-0.03	[-0.73; 0.67]	-0.01	[-0.09; 0.06]	0	[-0.09; 0.06]

Table S1: Average treatment effect with 95% CI for all subgroup analysis

Figure S3

Subgroup analysis: disaggregated age (55-64; 65-74; 75+) for circulatory mortality, intervention point 2003

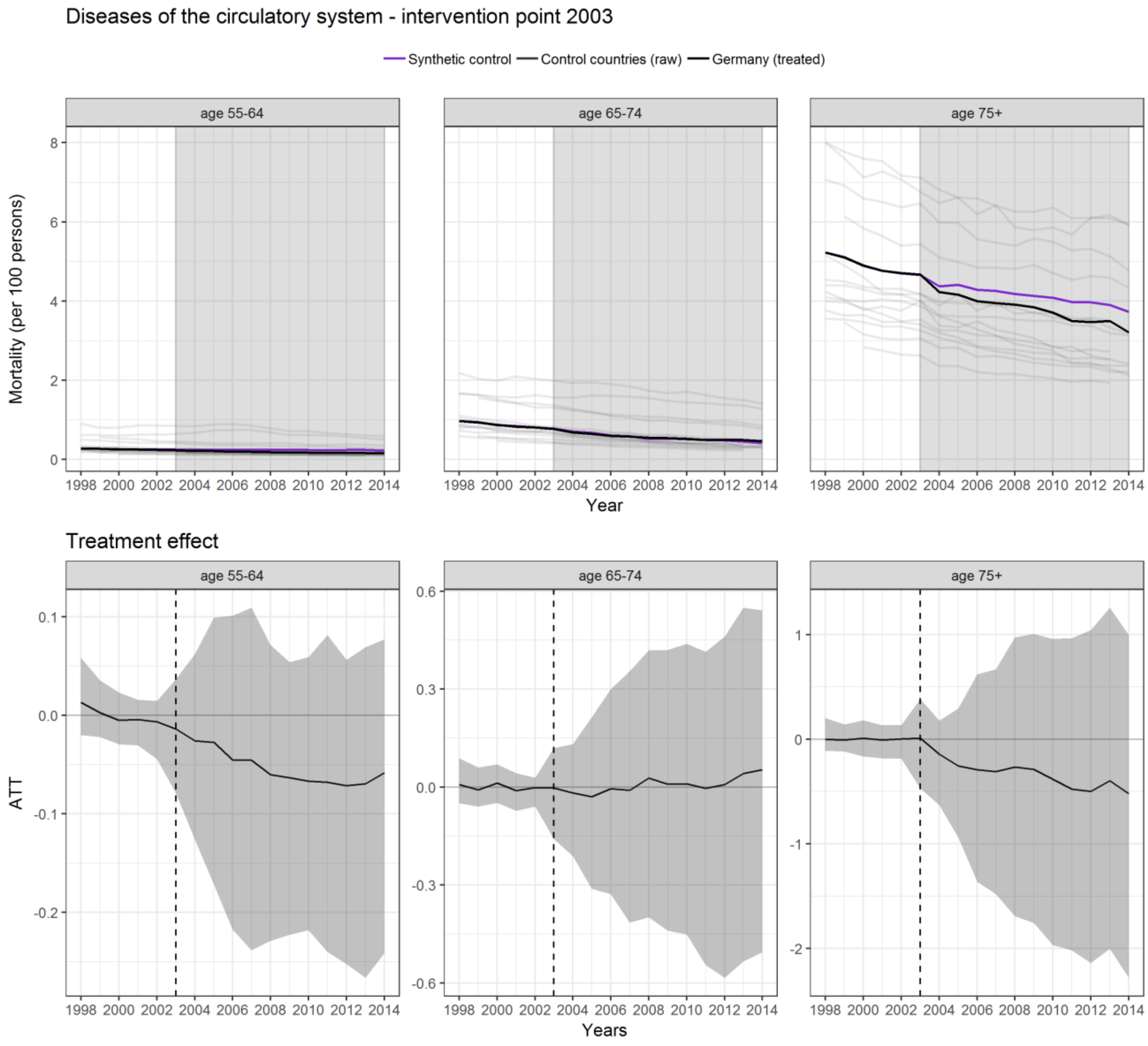

Figure S4

Subgroup analysis: disaggregated age (55-64; 65-74; 75+) for circulatory mortality, intervention point 2006

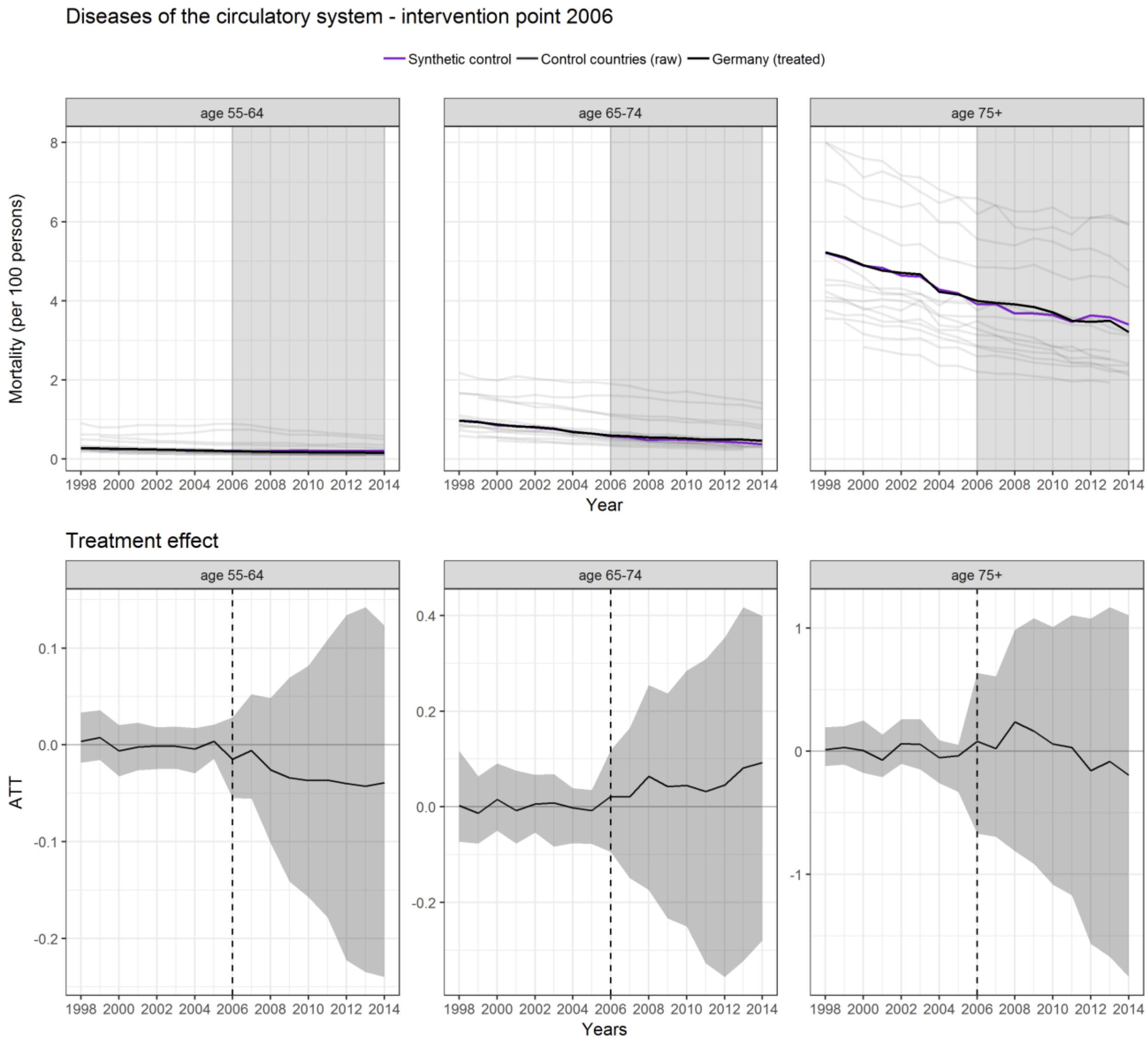

Figure S5

Subgroup analysis: disaggregated age (55-64; 65-74; 75+) for circulatory mortality, intervention point 2009

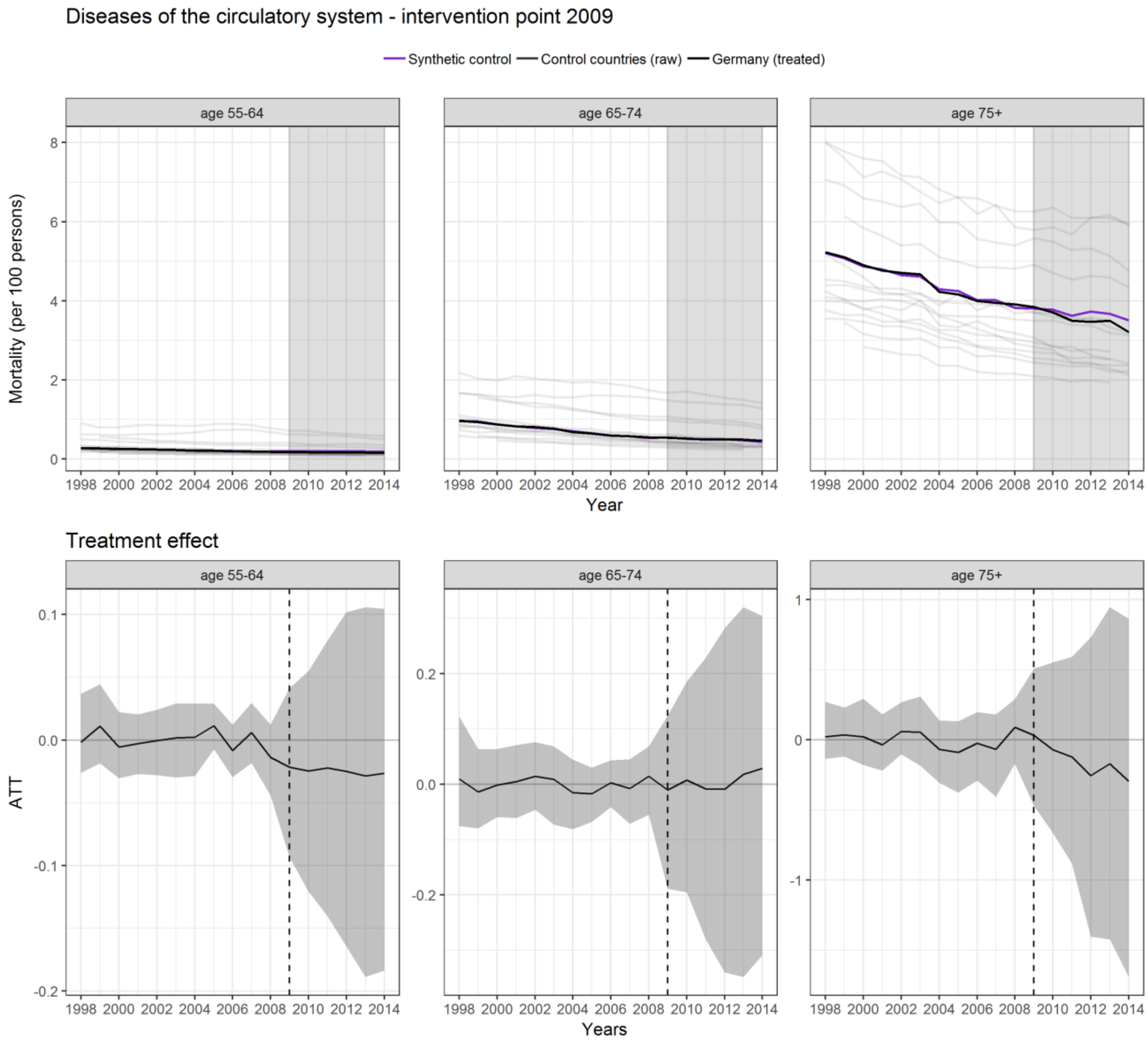

Figure S6

Subgroup analysis: disaggregated age (55-64; 65-74; 75+) for all-cause mortality, intervention point 2003

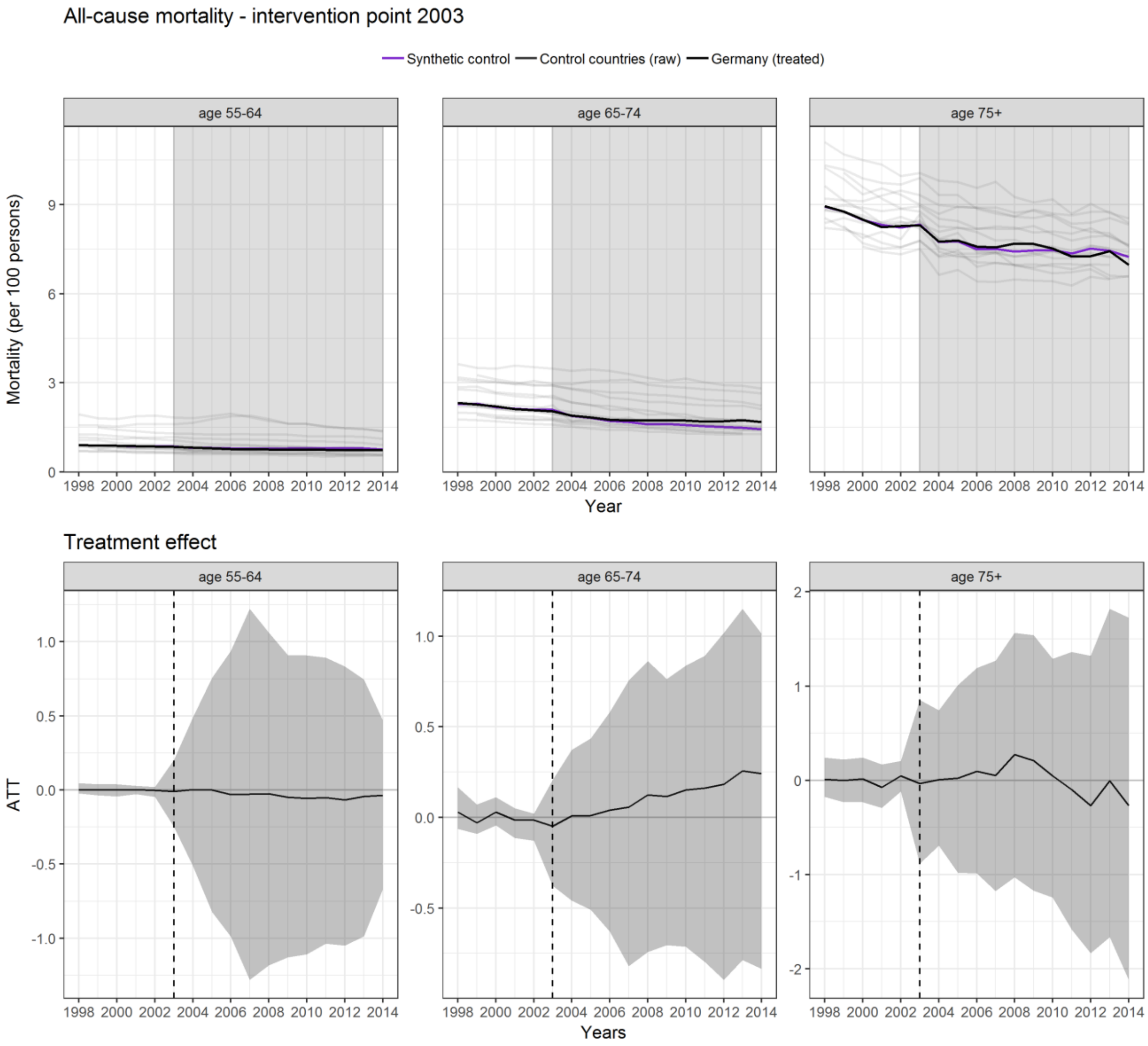

Figure S7

Subgroup analysis: disaggregated age (55-64; 65-74; 75+) for all-cause mortality, intervention point 2006

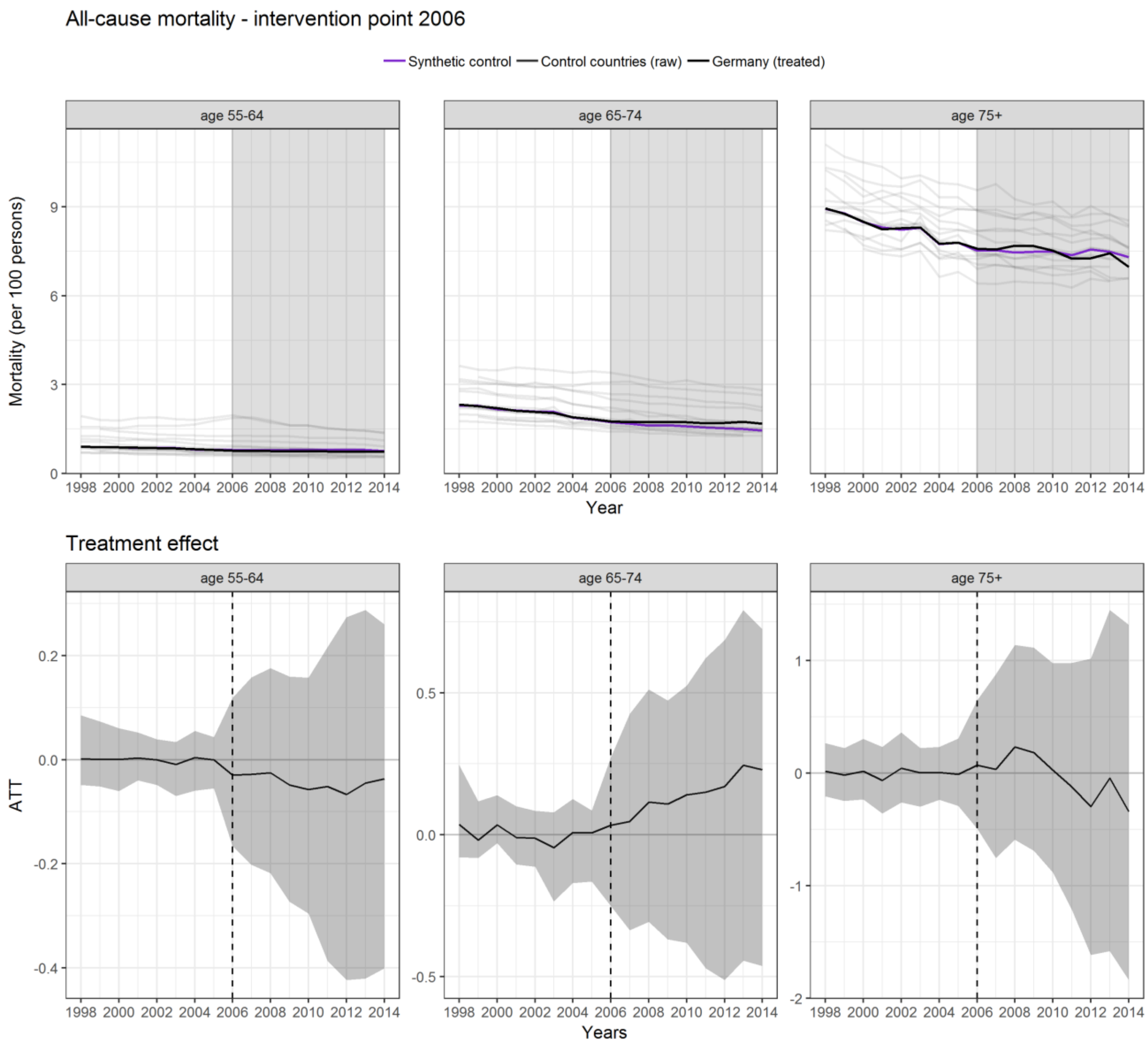

Figure S8

Subgroup analysis: disaggregated age (55-64; 65-74; 75+) for all-cause mortality, intervention point 2009

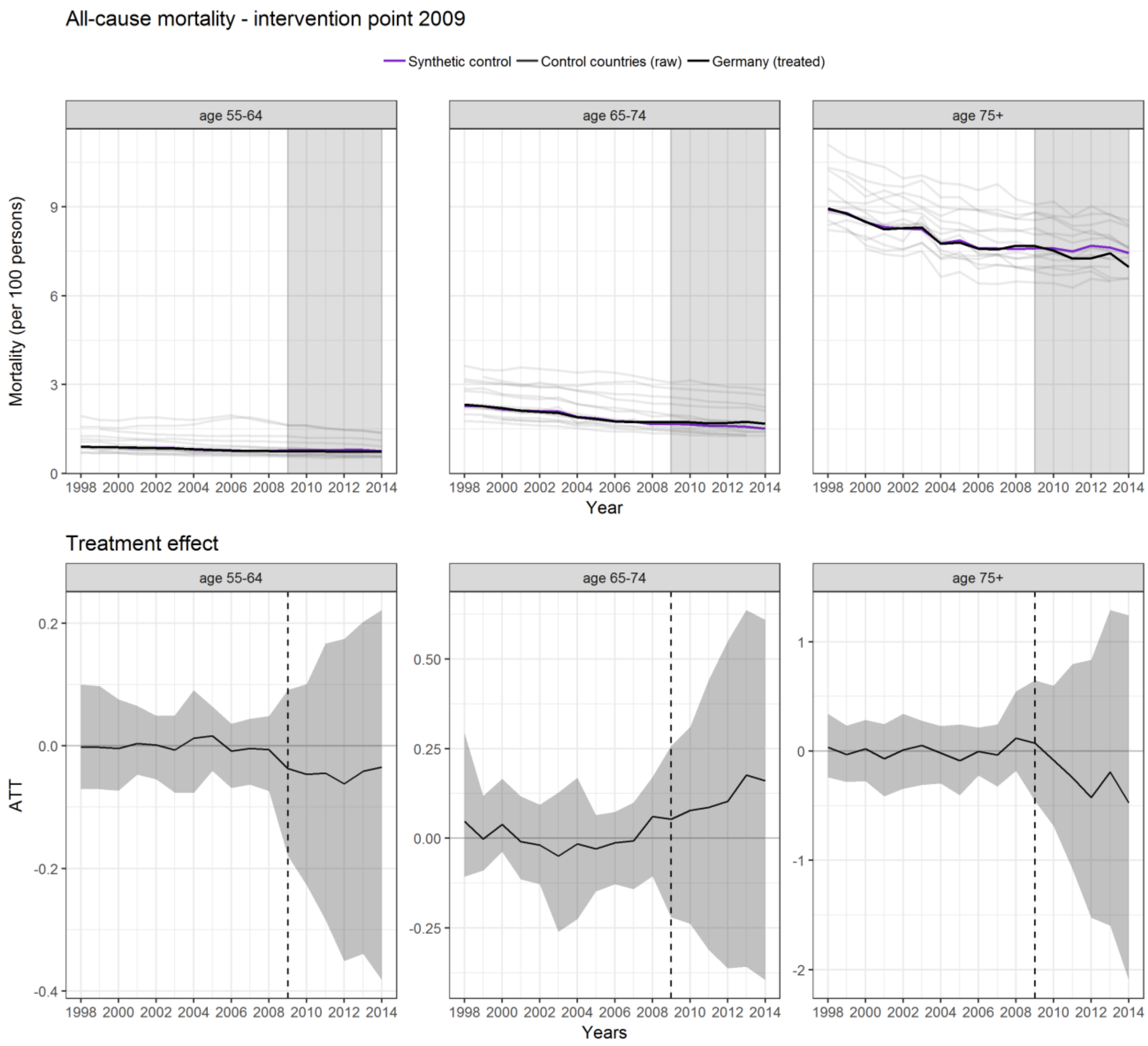

Figure S9

Sensitivity analysis: younger age group (20-54), where no or minimal effect is to be expected, for circulatory mortality.

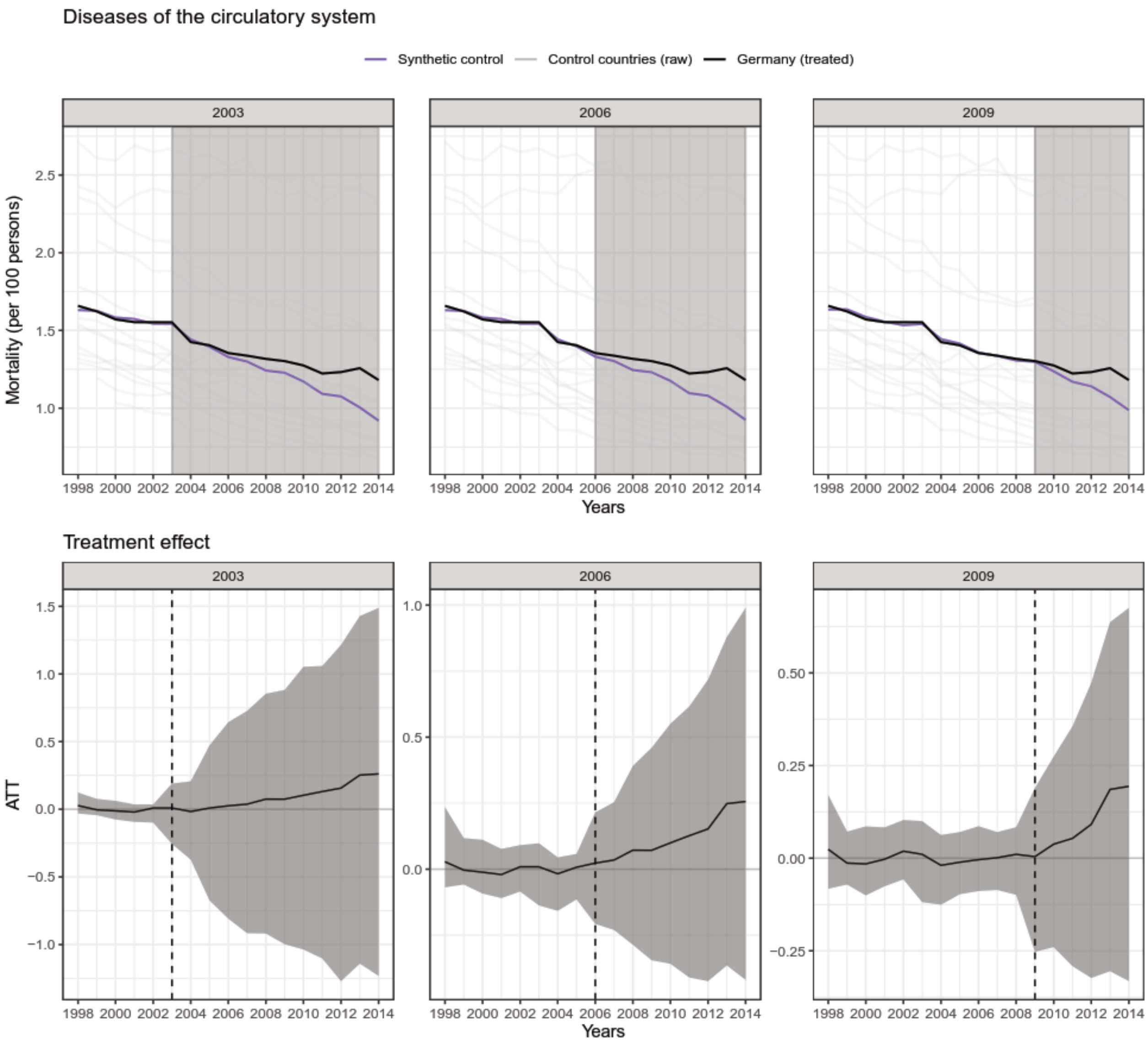

Figure S10
Sensitivity analysis: younger age group (20-54), where no or minimal effect is to be expected, for all-cause mortality.

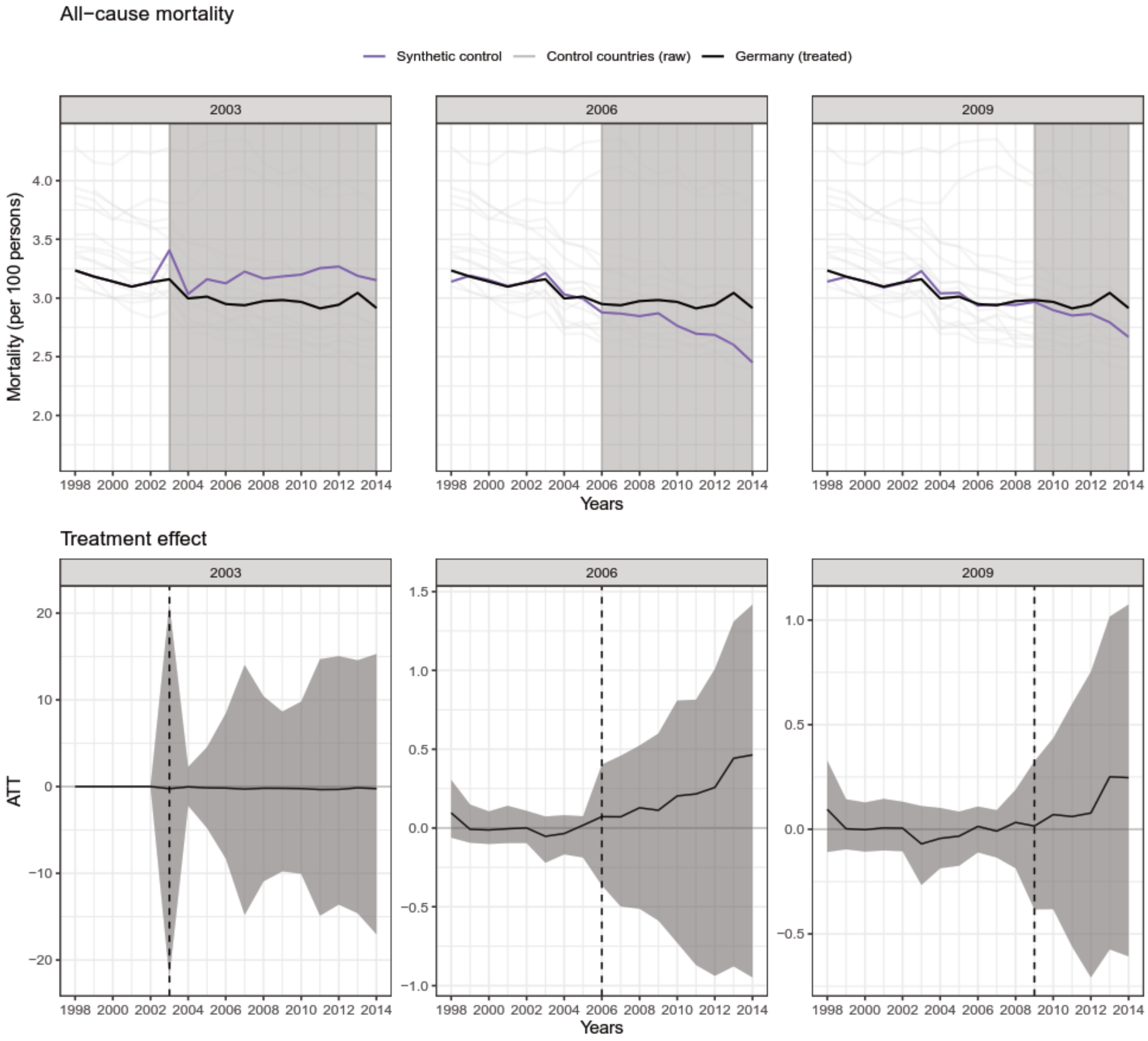

Figure S11

Sensitivity analysis: leave-one-country-out analyses for CHD mortality

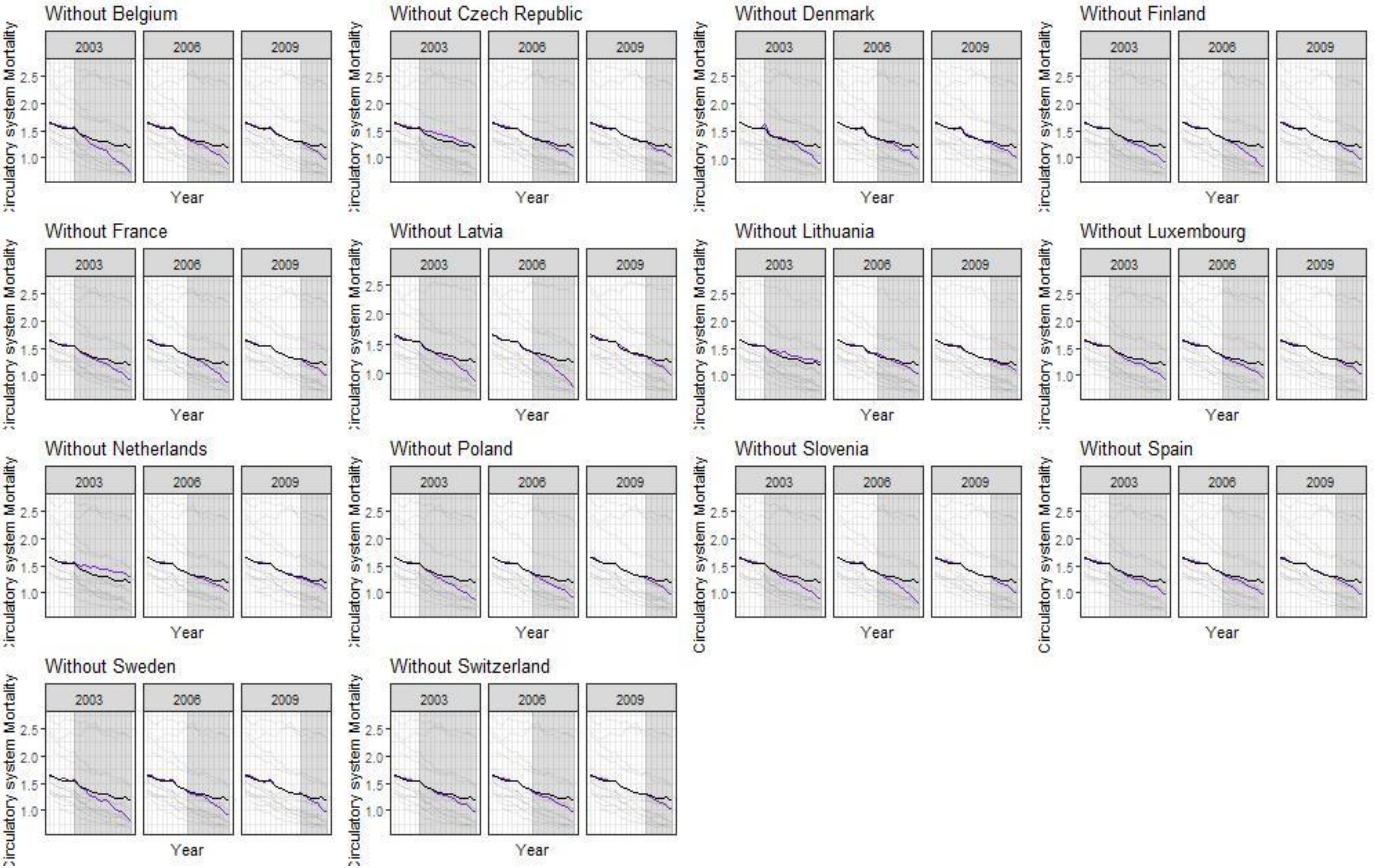

Figure S12

Sensitivity analysis: leave-one-country-out analyses for all-cause mortality

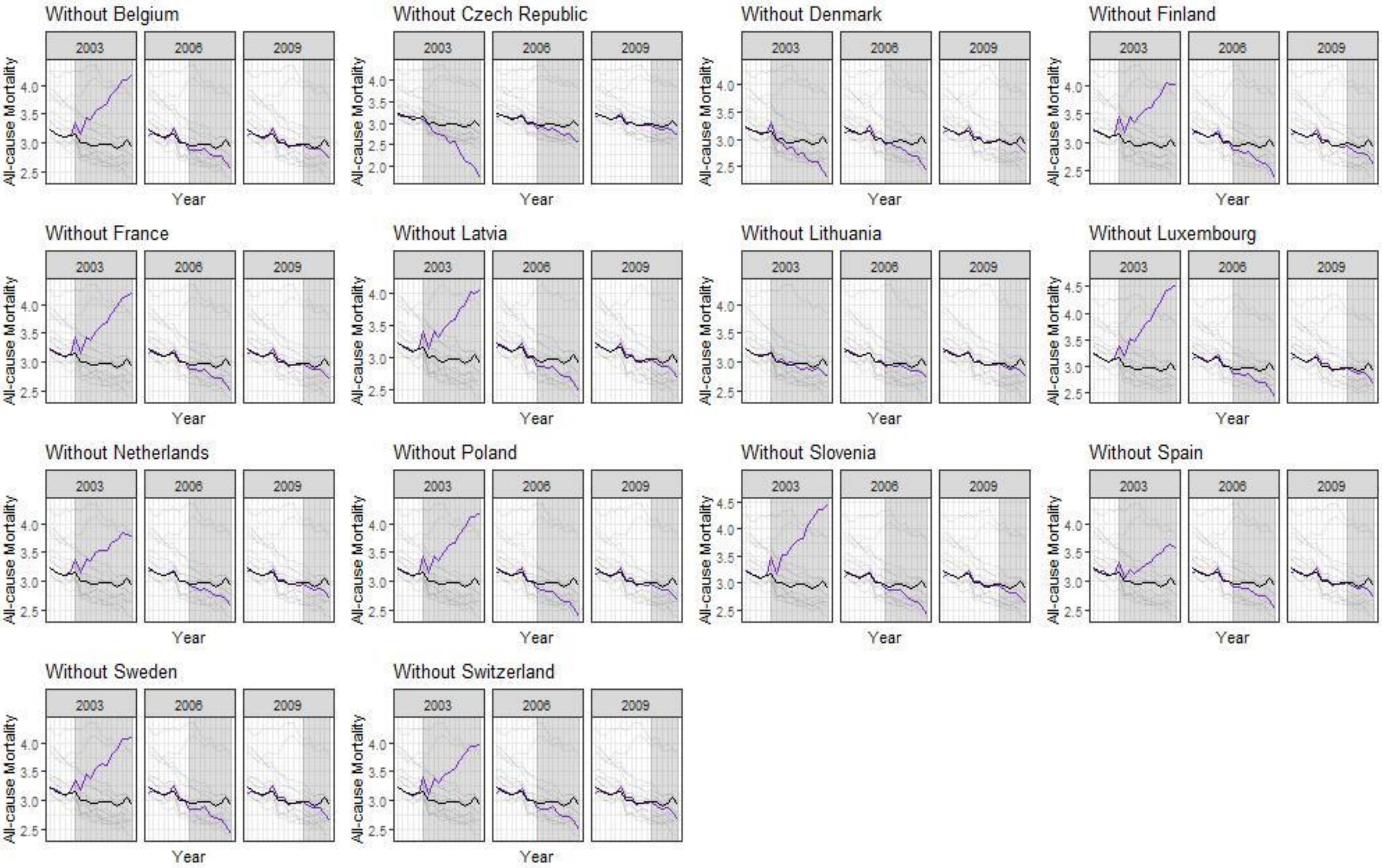

Supplement: Supplementary file 1 — Additional file 1 Fig. S1. Enrollment history for the German DMPs for type 2 diabetes and CHD. Table S1. Description of analyzed variables and the source of the data. Fig. S2. Trends in circulatory mortality (top panel) and all-cause mortality (bottom panel) in Germany as well as the rest of the European countries from 1998 to 2014. Table S2. The average treatment effects as well as the 95% confidence interval (CI), for each subgroup, each outcome and each intervention time point. Fig. S3. Subgroup analysis: disaggregated age (55–64; 65–74; 75+) for circulatory mortality, intervention point 2003. Fig. S4. Subgroup analysis: disaggregated age (55–64; 65–74; 75+) for circulatory mortality, intervention point 2006. Fig. S5. Subgroup analysis: disaggregated age (55–64; 65–74; 75+) for circulatory mortality, intervention point 2009. Fig. S6. Subgroup analysis: disaggregated age (55–64; 65–74; 75+) for all-cause mortality, intervention point 2003. Fig. S7. Subgroup analysis: disaggregated age (55–64; 65–74; 75+) for all-cause mortality, intervention point 2006. Fig. S8. Subgroup analysis: disaggregated age (55–64; 65–74; 75+) for all-cause mortality, intervention point 2009. Fig. S9. Sensitivity analysis: younger age group (20–54), where no or minimal effect is to be expected, for circulatory mortality. Fig. S10. Sensitivity analysis: younger age group (20–54), where no or minimal effect is to be expected, for all-cause mortality. Fig. S11. Sensitivity analysis: leave-one-country-out analyses for CHD mortality. Fig. S12. Sensitivity analysis: leave-one-country-out analyses for all-cause mortality. [file 12889_2021_12050_MOESM1_ESM.pdf]
